# Serum tau protein elevation in migraine: a cross-sectional case–control study

**DOI:** 10.1186/s10194-023-01663-5

**Published:** 2023-09-19

**Authors:** Lucas Hendrik Overeem, Bianca Raffaelli, Robert Fleischmann, Marie Süße, Antje Vogelgesang, Aleksandra Maleska Maceski, Athina Papadopoulou, Klemens Ruprecht, Wendy Su, Mirja Koch, Anke Siebert, Michal Arkuszewski, Nadia Tenenbaum, Jens Kuhle, Uwe Reuter

**Affiliations:** 1 Department of Neurology With Experimental Neurology, Charité – Universitätsmedizin Berlin, Charitéplatz 1, Berlin, 10117 Germany; 2 Doctoral Program, International Graduate Program Medical Neurosciences, Humboldt Graduate School, Berlin, 10117 Germany; 3 Clinician Scientist Program, Berlin Institute of Health (BIH), Berlin, 10117 Germany; 4 Department of Neurology, Universitätsmedizin Greifswald, Greifswald, 17475 Germany; 5 Department of Neurology, University Hospital and University of Basel, Basel, 4051 Switzerland; 6 Multiple Sclerosis Centre and Research Center for Clinical Neuroimmunology and Neuroscience (RC2NB), Departments of Biomedicine and Clinical Research, University Hospital and University of Basel, Basel, 4051 Switzerland; 7 Novartis Pharma AG, Basel, 4056 Switzerland; 8 EMD Serono Research and Development Institute, New York, NY USA

**Keywords:** Headache, CSF, Trigeminal Nerve, Migraine, Tau protein

## Abstract

**Background:**

Migraine is a disorder associated with neuropeptide release, pain and inflammation. Tau protein has recently been linked to inflammatory diseases and can be influenced by neuropeptides such as CGRP, a key neurotransmitter in migraine. Here, we report serum concentrations of total-tau protein in migraine patients and healthy controls.

**Methods:**

In this cross-sectional study, interictal blood samples from *n* = 92 patients with episodic migraine (EM), *n* = 93 patients with chronic migraine (CM), and *n* = 42 healthy matched controls (HC) were studied. We assessed serum total-tau protein (t-tau) and for comparison neurofilament light chain protein (NfL), glial fibrillary acidic protein (GFAP), and ubiquitin carboxy-terminal hydrolase L (UCH-L1) concentrations using the Neurology 4-plex kit, on a single molecule array HD-X Analyzer (Quanterix Corp Lexington, MA). Matched serum/cerebrospinal fluid (CSF) samples were used for post-hoc evaluations of a central nervous system (CNS) source of relevant findings. We applied non-parametric tests to compare groups and assess correlations.

**Results:**

Serum t-tau concentrations were elevated in EM [0.320 (0.204 to 0.466) pg/mL] and CM [0.304 (0.158 to 0.406) pg/mL] patients compared to HC [0.200 (0.114 to 0.288) pg/mL] (*p* = 0.002 vs. EM; *p* = 0.025 vs. CM). EM with aura [0.291 (0.184 to 0.486 pg/mL); *p* = 0.013] and EM without aura [0.332 (0.234 to 0.449) pg/mL; *p* = 0.008] patients had higher t-tau levels than HC but did not differ between each other. Subgroup analysis of CM with/without preventive treatment revealed elevated t-tau levels compared to HC only in the non-prevention group [0.322 (0.181 to 0.463) pg/mL; *p* = 0.009]. T-tau was elevated in serum (*p* = 0.028) but not in cerebrospinal fluid (*p* = 0.760). In contrast to t-tau, all proteins associated with cell damage (NfL, GFAP, and UCH-L1), did not differ between groups.

**Discussion:**

Migraine is associated with t-tau elevation in serum but not in the CSF. Our clinical study identifies t-tau as a new target for migraine research.

**Supplementary Information:**

The online version contains supplementary material available at 10.1186/s10194-023-01663-5.

## Introduction

Migraine is a highly prevalent primary headache disorder with a multitude of debilitating symptoms [1]. The pathophysiology of migraine is complex and multifaceted but it is known that one of the key anatomical structures, which is involved in pain generation, is the trigeminal nervous system [2]. Trigeminally mediated inflammation has been identified as a component in the pathophysiology of migraine [3–5]. Anti-inflammatory drugs such as cyclooxygenase (COX) inhibitors abort migraine attacks successfully and serotonin 5-HT_1B/D/F_ receptor agonists (triptans) block sterile neuro-inflammation in experimental animal models [6–10]. Nevertheless, a deeper understanding of the underlying molecular and pathophysiological mechanisms leading to migraine attacks is crucial for the development of more effective therapeutic strategies. This paper aims to explore the potential role of tau protein, a microtubule-associated protein crucial for neuronal stability and function i.e. axonal transport in migraine pathophysiology.

Neuropeptides play a crucial role in pain and inflammation. The dysregulation of certain neuropeptides such as neuropeptide Y in combination with abnormal tau proteins has been reported [11], but the direct link between tau and these neuropeptide abnormalities remains to be elucidated. Studies show that misfolded or phosphorylated tau proteins have the capacity to induce neuroinflammation and initiate the breakdown of the bloodbrain barrier [12–16]. For example, substance P reduced tau phosphorylation in a rat animal model of concussion, which could be reverted by a NK1 receptor antagonist [17]. Microtubule alterations could therefore potentially contribute to neurological disorders linked with inflammation including migraine or cluster headache. In line, a recent study showed that tau protein is increased in inflammatory neuropathies such as Guillain–Barre syndrome [18, 19].

Another central neuropeptide in the pathogenesis of migraine is Calcitonin Gene-Related Peptide (CGRP) [20]. It is a potent vasodilator and pain signal transmitter. In migraine, microtubule instability could alter axonal function and affect the release of CGRP from peripheral trigeminal neurons [21, 22]. The possibility of a role of tau-related microtubule stability in CGRPmediated disorders represents an interesting target for investigation. In fact, an experimental animal study showed that CGRP receptor antagonists have effects on neuroinflammation in tau-mediated disorders [23]. Additionally, CGRP was found to inhibit tau hyperphosphorylation and thereby reduced total tau levels in a focal cerebral ischemia/reperfusion model [24]. Along another line of evidence, CGRP is elevated during Cortical Spreading Depression (CSD) a wave of neuronal and glial depolarization that is thought to be the underlying pathophysiological correlate of migraine aura [25, 26]. The role of tau in neuroinflammation has been described in tauopathies, for example, misfolded or phosphorylated tau might have different capacities to induce neuroinflammation [12–14].

Based on the role of tau in maintaining neuronal structure and potentially influencing neuronal function, it is possible that tau dysregulation could affect CSD and consequently influence CGRP release, thus contributing to migraine pathophysiology.

Pituitary adenylate cyclase-activating polypeptide (PACAP) is also implicated in migraine pathophysiology as initially proposed by Schytz et al. [27], and recently confirmed in a phase II clinical trial (NCT05133323). In this study for the prevention of migraine the antibody Lu AG09222, which blocks PACAP, effectively reduced monthly migraine days superior to placebo [27, 28]. The PAC-1 receptor, a binding site of PACAP, is expressed in the human trigeminal ganglion as well as in the brain [29]. Interestingly, the activation of the same receptor (PAC-1) prevents the accumulation of aggregate-prone tau in transgenic tauopathy mice brain pointing to a role of the PAC-1 receptor in both diseases [30].

Most importantly, tau protein dysregulation is a well-established factor in several neurodegenerative disorders such as Alzheimer´s disease, but several other markers for neurodegeneration such as neurofilament light chain (NfL) [31], glial fibrillary acidic protein (GFAP) [32], and ubiquitin carboxy-terminal hydrolase L1 (UCH-L1) exist [33].

The exploration of the link between tau and migraine could help better understand the pathophysiology of this disease. This investigation aims to explore tau protein serum levels in the whole spectrum of patients with migraine in a cross-sectional case-control study. To assure that potential tau elevation in migraine is not a consequence of neuronal cell damage, a phenomenon that is typically associated with tau increase, we also assessed typical markers of neurodegeneration in this study. Additional serum and CSF samples were used for the evaluation of a central or peripheral origin of elevated biomarkers.

## Materials and methods

### Design

This is an observational cross-sectional study with a case–control design, which was approved by the ethical committee of Charité Universitätsmedizin Berlin (EA4/149/18). Written informed consent was obtained from all participants before study inclusion.

The study consisted of three study groups: individuals with episodic migraine (EM), with chronic migraine (CM), and healthy controls (HC). The migraine groups were recruited from the headache outpatient clinic of Charité – Universitätsmedizin Berlin between June 2019 and February 2021. All patients have been diagnosed according to the ICHD-3 guidelines [34]. We recruited healthy control participants using the intranet platform of Charité Universitätsmedizin Berlin and notice boards.

### Inclusion & exclusion criteria

Patients were eligible if they had an existing ICHD-3 diagnosis for EM or CM, with or without aura with an age between 18 and 65 years. Patients were excluded if they suffered from any other neurological, psychiatric, or other chronic or inflammatory diseases or other primary headache disorder with the exception of infrequent episodic tension-type headache.

While we included CM patients with and without prophylaxis, in the EM group migraine prophylactic medications were not allowed, regardless of indication. In detail, the following medications led to study exclusion in the EM group: Metoprolol, Propranolol, Bisoprolol, Amitriptyline, Topiramate, Candesartan, Flunarizine, a CGRP monoclonal antibody, or a CGRP-receptor monoclonal antibody [35].

Healthy controls were eligible if they were between 18 and 65 years of age and if they did not suffer from any headache disorders or any other neurological disease. A positive family history of migraine, dementia, any chronic disease, a history of stroke or head trauma, or a history of or present moderate or severe depression as determined by the Beck Depression Inventory led to exclusion. The occurrence of any headache in the past three months also led to exclusion.

### Assessment

For blood sampling, participants (including healthy controls) were in a non-fasting state and had to be free of any headache at the time of the blood withdrawal. The last headache attack of participants with migraine had to be completely resolved at least 12 h prior to blood withdrawal. Blood samples were taken from each participant using a 5 mL serum tube containing CAT Serum Sep Clot Activator (VACUETTE: 456010) from the cubital vein. Samples were centrifuged after blood withdrawal for 10 min at 800g at room temperature. The serum was stored within two hours at -80°C until sample analysis. We analyzed t-tau, NfL, GFAP, and UCH-L1 concentrations in serum using the Neurology 4-plex assay (HD‐1/HD‐X Item 102153) (Quanterix Corp, Lexington, MA) on a single molecule array (Simoa®) HD-X Analyzer (Quanterix Corp) at the Departments of Biomedicine and Clinical Research, University Hospital and University of Basel, Basel, Switzerland. Values under the limit of detection (LOD) of t-tau, NfL, GFAP, and UCH-L1 were set to 0.007, 0.025, 0.042, and 0.120 pg/ml, respectively. Inter-assay mean coefficient of variation (CV) was established with one serum and two assay kit controls. The mean CVs were 7.4%, 2.8%, 3.2% and 10.7% for t-tau, NfL, GFAP and UCH-L1 respectively.

### Outcomes

The primary endpoint of the study was the difference in the concentrations of t-tau, NfL, GFAP, and UCH-L1 between the HC group and both migraine (EM/CM) groups. Secondary endpoints were the differences of t-tau, NfL, GFAP, and UCH-L1, across HC and EM with (EMA) and without aura (EMO), as well as between HC and CM with (CM +)/ without (CM-) prophylactic treatment. Further secondary endpoints were the correlations between the biomarkers and the demographic variables age and body mass index (BMI) and variables related to headaches: days since resolution of the last headache attack, days since last migraine attack, monthly headache days and monthly migraine days.

### Variables

Data for monthly headache days (MHD), monthly migraine days (MMD), days since the last headache/migraine day, and monthly days with acute medication (AMD) were collected from the headache diaries. These variables were determined on the 28-day period before blood collection.

A migraine headache was defined as a headache with or without aura lasting for at least 30 min meeting at least one of the following criteria [34]: (A) ≥ 2 of the following pain features: unilateral, throbbing, moderate to severe, exacerbated by exercise/physical activity; (B) ≥ 1 of the following associated symptoms: nausea and/or vomiting, and photophobia and phonophobia; (C) Intake of migraine-specific acute medication, which was effective. We also collected the demographic characteristics of age, sex, ethnicity, body mass index (BMI; kg/m2), and alcohol and tobacco consumption.

### CSF/serum analysis

In a post-hoc analysis, we assessed matched CSF/serum samples. These were acquired during routine in-hospital diagnostic work-up of headache disorders (patients eventually diagnosed with migraine) and after exclusion of CNS pathology in psychosomatic disorders (controls) at the Greifswald University Medicine, Germany. The control group patients did not suffer from any headache. Patients provided written informed consent for their samples to be used for research purposes. The use of the biospecimens for headache research was approved by the Ethical Committee, Greifswald University Medicine, Germany (BB 161/18). All samples were stored at -80°C within 24 h after sample collection. Patients were diagnosed with migraine according to ICHD-3 criteria and had no other diagnosis of any systemic chronic or neurological diseases. The samples from migraine patients were taken during the diagnostic work up (*n* = 23) and from controls (*n* = 16). Samples were analyzed for t-tau, NfL, GFAP, and UCH-L1 concentrations in serum and CSF using the Neurology 4-plex assay (SR-X Item 102,153) (Quanterix Corp, Lexington, MA) on a single molecule array (Simoa®) SR-X Analyzer (Quanterix Corp) at the Greifswald University Medicine.

### Sample-size and statistical analysis

Due to the novelty of this approach, we did not calculate the sample size a priori for the comparison of serum t-tau and therefore our current sample size was determined by convenience. The biomarker values are reported as median (*Md*) interquartile range (*IQR*), other continuous variables are reported as mean ± standard deviation (*SD*), and counts and percentages are used for categorical variables. Statistical analyses were carried out using SPSS 28.0.1.0 (IBM Corp., Armonk, NY, USA). To test the normal distribution of our data, we used the Kolmogorov–Smirnov Test. Since our data remained non-normal after log-transformation, we chose a nonparametric approach. To compare groups, we used the Independent-Samples Kruskal–Wallis Test with a Dunn’s Post-Hoc test with Bonferroni correction for multiple comparisons. For post-hoc analyses of matched CSF/serum samples, we used the Mann–Whitney test (unpaired for the hypothesis of confirmation of findings). To correct for the covariates age, sex, and BMI, we used a Quade non-parametric ANCOVA.

Spearman's rho test was used for the testing of correlations. To analyze whether these biomarkers are able to distinguish between HC and migraine patients, receiver operating characteristics (ROC) statistics and area under the curve (AUC) values were calculated. A p-value below 0.05 was considered statistically significant.

### Data availability

The data that support the findings of this study are available from the corresponding author, upon reasonable request.

## Results

### Characteristics of the study population

For this study, *n* = 288 participants were screened. Forty-four participants did not meet the eligibility criteria and 17 participants did not sign informed consent. Data from the remaining 227 participants (*n* = 42 healthy controls, *n* = 92 patients with EM, and *n* = 93 patients with CM) were included for analyses. Age, gender, and BMI did not differ between groups (for all *p* > 0.05). Migraine patients were more likely to consume less alcohol compared to healthy controls (*p* < 0.001). Tobacco consumption did not differ between groups (*p* = 0.119), Table 1. The characteristics of our subgroups can be found in Supplementary Table 1.

**Table 1:** Clinical and demographic characteristics of healthy controls, episodic migraine, and chronic migraine

	**Healthy controls *n*= 42**	**Episodic migraine *n* = 92**	**Chronic migraine *n* = 93**	***P***-value^a^
**General**				
Age in years	44.6 ± 12.0	41.7 ± 10.6	45.1 ± 12.1	0.071
Female, n (%)	35 (83.3)	73 (79.3)	80 (86.0)	0.485
BMI, mean ± SD	24.4 ± 4.5	24.0 ± 3.8	24.2 ± 4.1	0.990
Ethnicity				1.000
Caucasian	41 (97.6)	91 (98.9)	92 (98.9)	
Other	1 (2.4)	1 (1.1)	1 (1.1)	
Alcohol consumption, n (%)				< 0.001*
Never (rarely)	2 (4.8)	33 (35.9)	53 (57.0)	
< Monthly	6 (14.3)	18 (19.6)	6 (6.5)	
Monthly	14 (33.3)	22 (23.9)	19 (20.4)	
Weekly	19 (45.2)	18 (19.6)	15 (16.1)	
Daily	1 (2.4)	1 (1.1)	0	
Tobacco consumption, n (%)				0.329
Never	25 (59.5)	68 (73.9)	69 (74.2)	
Past	13 (31.0)	15 (16.3)	16 (17.2)	
Current	4 (9.5)	9 (9.8)	8 (8.6)	
**Headache characteristics**				
Migraine aura	-	43 (46.7)	45 (48.4)	-
Prophylactic use	-	0	45 (48.4)	-
Total headache days	-	7.3 ± 3.2	15.0 ± 7.5	< 0.001*
Total migraine days	-	6.2 ± 3.0	12.2 ± 6.7	< 0.001*
Total acute medication days	-	5.4 ± 2.9	8.6 ± 6.5	< 0.001*
Days since last migraine attack	-	5.6 ± 6.4	2.1 ± 3.1	< 0.001*
Days since last headache day	-	4.5 ± 5.5	1.7 ± 2.8	< 0.001*

*BMI* Body mass index, calculated as kg/m^2^ where kg is a person's weight in kilograms and m^2^ is their height in meters squared; *SD* Standard deviation

Continuous values are expressed as mean ± SD

^a^p-value for between-group difference, Chi^2^ test was used for categorical variables and Independent-Samples Kruskal–Wallis Test or Mann–Whitney Test for continuous variables

^*^p-value < 0.05

On average (SD), EM patients had 6.2 (3.0) monthly migraine days, while CM patients had 12.2 (6.7) monthly migraine days. Further headache characteristics are shown in Table 1.

The matched CSF/serum samples (*n* = 39) were mostly from females (83%), with a mean age of 37.4 ± 13.1 years.

### Episodic and chronic migraine

**Fig. 1:**
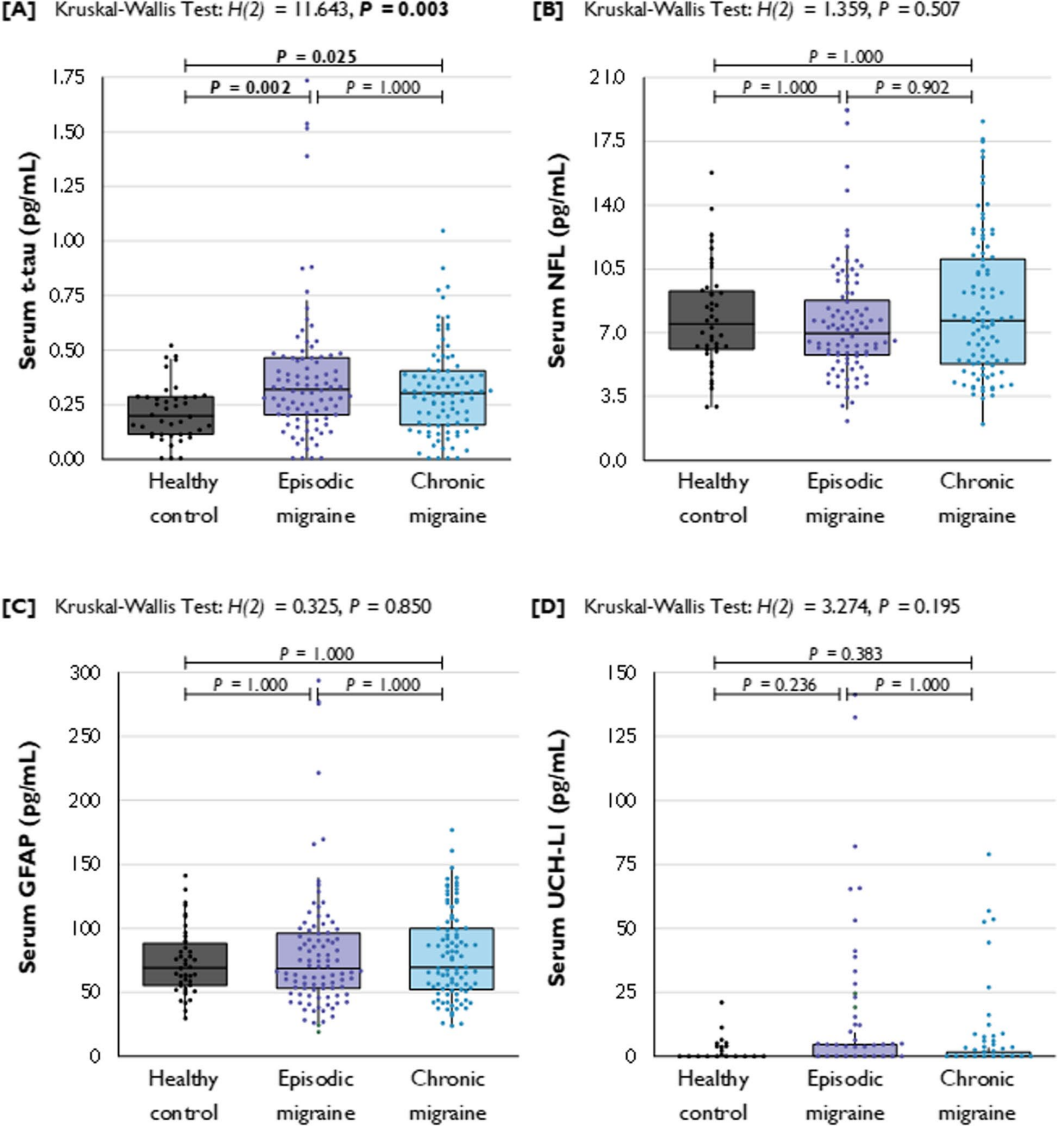
Blood serum concentrations of total-tau, NfL, GFAP, and UCH-L1 in pg/mL for patients with EM and CM and healthy controls. Legend: t-tau = total tau; NfL = neurofilament-light; GFAP = glial fibrillary acidic protein; UCH-L1 = ubiquitin C-terminal hydrolase L1; HCs = healthy controls; EM = episodic migraine; CM = chronic migraine. ^a^ *p*-value for between-group difference as estimated with the Independent-Samples Kruskal–Wallis Test for continuous variables, corrected with the Bonferroni correction for multiple comparisons. *P*-values < 0.05 are depicted in bold

Serum t-tau concentrations were different across HC, EM, and CM patients, *H(2)* = 11.643, *p* = 0.003, Fig. 1. Serum t-tau concentrations were higher in blood samples of EM patients (*Md* = 0.320 [0.204 to 0.466] pg/mL) and CM patients (*Md* = 0.304 [0.158 to 0.406] pg/mL) compared to HC (*Md* = 0.200 [0.114 to 0.288] pg/mL). Pairwise comparisons showed a significant difference between HC and EM (*p* = 0.002, *r* = 0.289) and HC and CM (*p* = 0.025, *r* = 0.230). T-tau levels from patients with EM and CM did not differ from each other (*p* = 1.000, *r* = 0.071), Table 2.

**Table 2:** Serum t-tau, NfL, GFAP, and UCH-L1 concentrations for HC, EM, and CM

Biomarker	Healthy control *n* = 42	Episodic migraine *n* = 92	Chronic migraine *n* = 93	*p*-value ^a^	Multiple variable adjusted *p*-value ^b^
t-tau	0.200 (0.114 to 0.288)	0.320 (0.204 to 0.466) ^**c**^	0.304 (0.158 to 0.406) ^**c**^	0.003*	0.002*
NfL	7.5 (6.0 to 9.4)	7.0 (5.7 to 8.9)	7.7 (5.2 to 11.1)	0.507	0.920
GFAP	69.4 (54.7 to 89.7)	68.5 (52.7 to 96.6)	69.6 (52.0 to 100.2)	0.850	0.757
UCH-L1	0.120 (0.120 to 0.357)	0.120 (0.120 to 4.693)	0.120 (0.120 to 1.824)	0.195	0.184

Values are expressed as median (interquartile range) in pg/mL

^a^*P*-values are estimated with an independent-samples Kruskal–Wallis test with a Dunn’s post-hoc test with Bonferroni correction for multiple comparisons

^b^*P*-values are estimated with a Quade nonparametric ANCOVA adjusting for age, sex, and body mass index

^*^p-value < 0.05

^**c**^Indicates statistical elevation vs. controls

No statistically significant differences of NfL, GFAP, and UCH-L1 were observed across HC, EM, and CM patients (*p* = 0.507, *p* = 0.850, and *p* = 0.195, respectively). The results are shown in Fig. 1. Serum concentrations of the biomarkers are illustrated in Table 2.

### Episodic migraine with and without aura

Stratification in EM for aura revealed differences in serum t-tau concentrations across HC, EMO (*n* = 49), and EMA (*n* = 43), *H(2)* = 11.180, (*p* = 0.004). Serum t-tau concentrations were higher in migraine patients with and without aura (*Md* = 0.332 [0.234 to 0.449] pg/mL for EMO and *Md* = 0.291 [0.184 to 0.486] pg/mL for EMA) in comparison to HC (*Md* = 0.200 [0.114 to 0.288] pg/mL). Pairwise comparisons showed significant differences between HC and EMO (*p* = 0.008, *r* = 0.315) and HC and EMA (*p* = 0.013, *r* = 0.305. These differences remained significant after the correction for age, sex, and BMI (*p* = 0.001 and *p* = 0.003, respectively) but not between patients with EMO and EMA (*p* = 1.000, *r* = 0.004). Our analysis did not find any differences for NfL, GFAP, and UCH-L1 between groups, Table 3a.

**Table 3:** Serum t-Tau, NfL, GFAP, and UCH-L1 concentrations for HC, EM, and CM

[A] Biomarker	Healthy control (HC) *n* = 42	*Episodic migraine without aura (EMO) n = 49*	*Episodic migraine with aura (EMA) n = 43*	*p*-values ^a^	Multiple variable adjusted *p*-value^b^
t-tau	0.200 (0.114 to 0.288)	0.332 (0.234 to 0.449)^**c**^	0.291 (0.184 to 0.486)^**c**^	0.004*	0.002*
NfL	7.5 (6.0 to 9.0)	7.2 (5.9 to 8.0)	6.6 (5.4 to 10.0)	0.637	0.769
GFAP	69.4 (54.7 to 90.0)	66.8 (53.0 to 95.0)	70.3 (48.5 to 97.0)	0.965	0.696
UCH-L1	0.120 (0.120 to 0.357)	0.120 (0.120 to 5.17)	0.120 (0.120 to 4.645)	0.210	0.150
**[B] Biomarker**	**Healthy control (HC) n = 42**	**Chronic migraine without prophylaxis (CM-) *n* = 48**	**Chronic migraine with prophylaxis (CM +) *n* = 45**	***p*-values**^**a**^	**Multiple variable adjusted *p*-value**^**b**^
t-tau	0.200 (0.114 to 0.288)	0.322 (0.181 to 0.463)^**c**^	0.293 (0.126 to 0.375)	0.012*	0.015*
NfL	7.5 (6.0 to 9.0)	7.8 (5.4 to 12.0)	7.3 (5.1 to 11.0)	0.693	0.201
GFAP	69.4 (54.7 to 90.0)	78.2 (56.7 to 106.0)	65.8 (49.0 to 100.0)	0.490	0.313
UCH-L1	0.120 (0.120 to 0.357)	0.241 (0.120 to 2.446)	0.120 (0.120 to 1.025)	0.083	0.104

Values are expressed as median (interquartile range) in pg/mL

^a^*P*-values are estimated with an independent-samples Kruskal–Wallis test with a Dunn’s post-hoc test with Bonferroni correction for multiple comparisons

^b^*P*-values are estimated with a Quade nonparametric ANCOVA adjusting for age, sex, and body mass index

^*^p-value < 0.05

^**c**^Indicates statistical elevation vs. controls

T-tau was correlated with BMI only in the subgroup of patients with episodic migraine without aura (*ρ* = -0.307, *p* = 0.032, *n* = 49). There was no association between t-tau and any of the following parameters in the EM group: age, number of monthly headache days, and monthly migraine days.

### Chronic migraine with (CM +) and without (CM-) prophylaxis

The stratification of CM patients according to current prophylactic treatment (no prophylaxis: CM-; *n* = 48 vs prophylaxis: CM + ; *n* = 45), revealed differences in t-tau concentrations across HC, CM-, and CM + , *H(2)* = 8.838, *p* = 0.012. T-tau was elevated in CM- patients (*Md* = 0.322 [0.181 to 0.463]) compared with HC (*Md* = 0.200 [0.114 to 0.288]) (*p* = 0.009, *r* = 0.307). This difference remained significant after the adjustment for age, sex, and BMI (*p* = 0.004). There was no difference of serum t-tau concentrations between CM + (*Md* = 0.293 [0.126 to 0.375]) patients and HC (*p* = 0.289, *r* = 0.184). Patients with CM- and CM + were not different from each other (*p* = 0.576, *r* = 0.140). Our analysis did not show any differences for NfL, GFAP, and UCH-L1 between groups, Table 3b.

### Correlations of t-tau with headache characteristics

For an unbiased analysis, we included solely migraine patients without prophylactic treatment in the assessment of the association between serum markers and headache characteristics (*n* = 140). Complete headache dairies were available from *n* = 122 (87%) patients. We did not find correlations between t-tau and the frequency of headache days or migraine days, and time between sample collection and last migraine day. Also, the number of days with acute medication was not correlated with t-tau.

### ROC curve analysis of t-tau

**Fig. 2:**
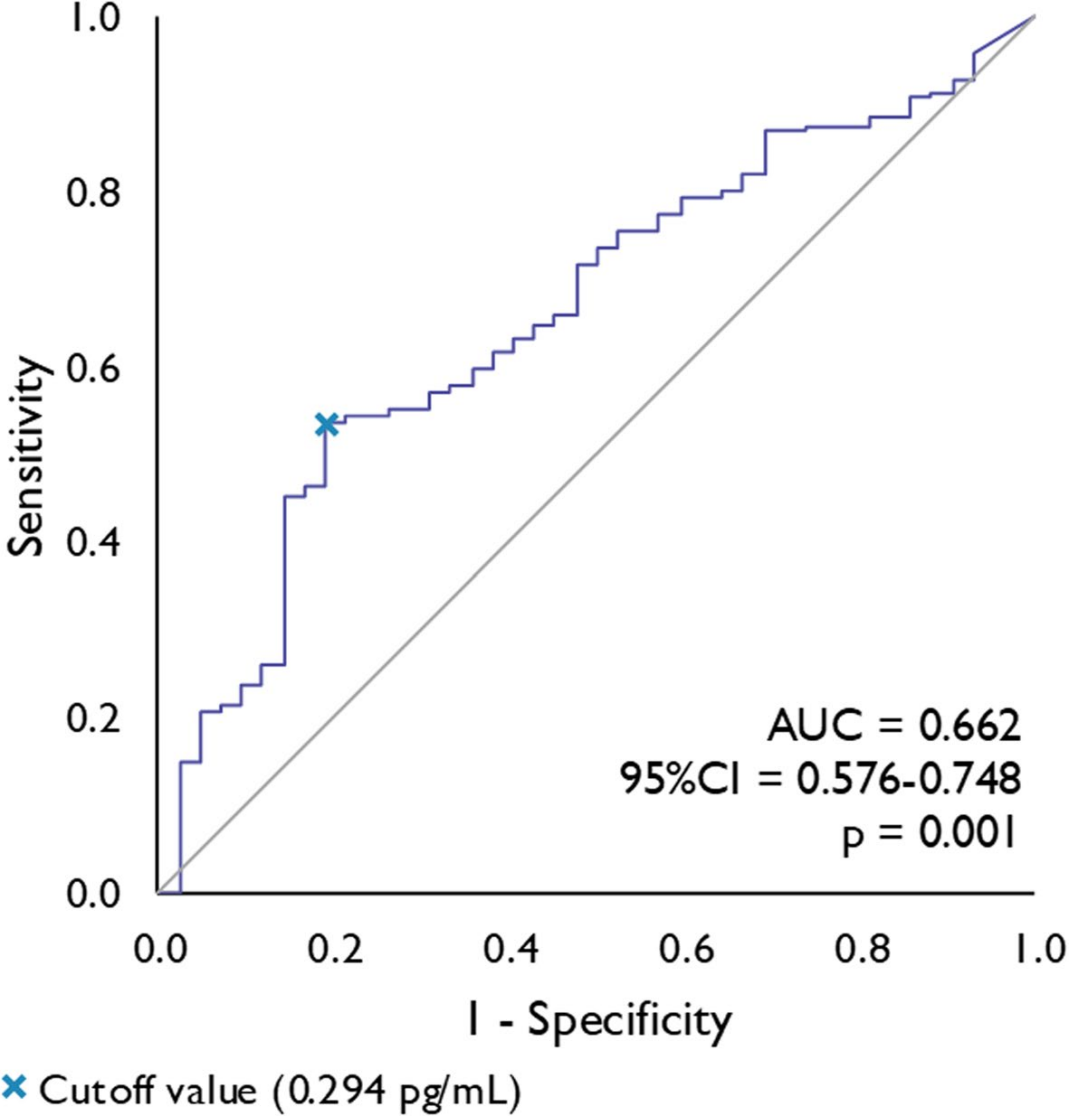
Receiver Operating Characteristics (ROC) curve analysis of serum total-tau concentrations for the prediction of migraine. Legend: AUC = area under the curve, CI = confidence interval

In order to assess whether t-tau levels can help to distinguish between migraine patients and healthy controls we performed ROC analysis. The data show that t-tau protein is able to distinguish between patients with migraine and healthy controls in our study at a cut-off value of 0.294 pg/ml (*p* < 0.001) with a sensitivity of 54% and a specificity of 81%, Fig. 2.

### Post-hoc analyses of matched CSF/serum samples

**Fig. 3:**
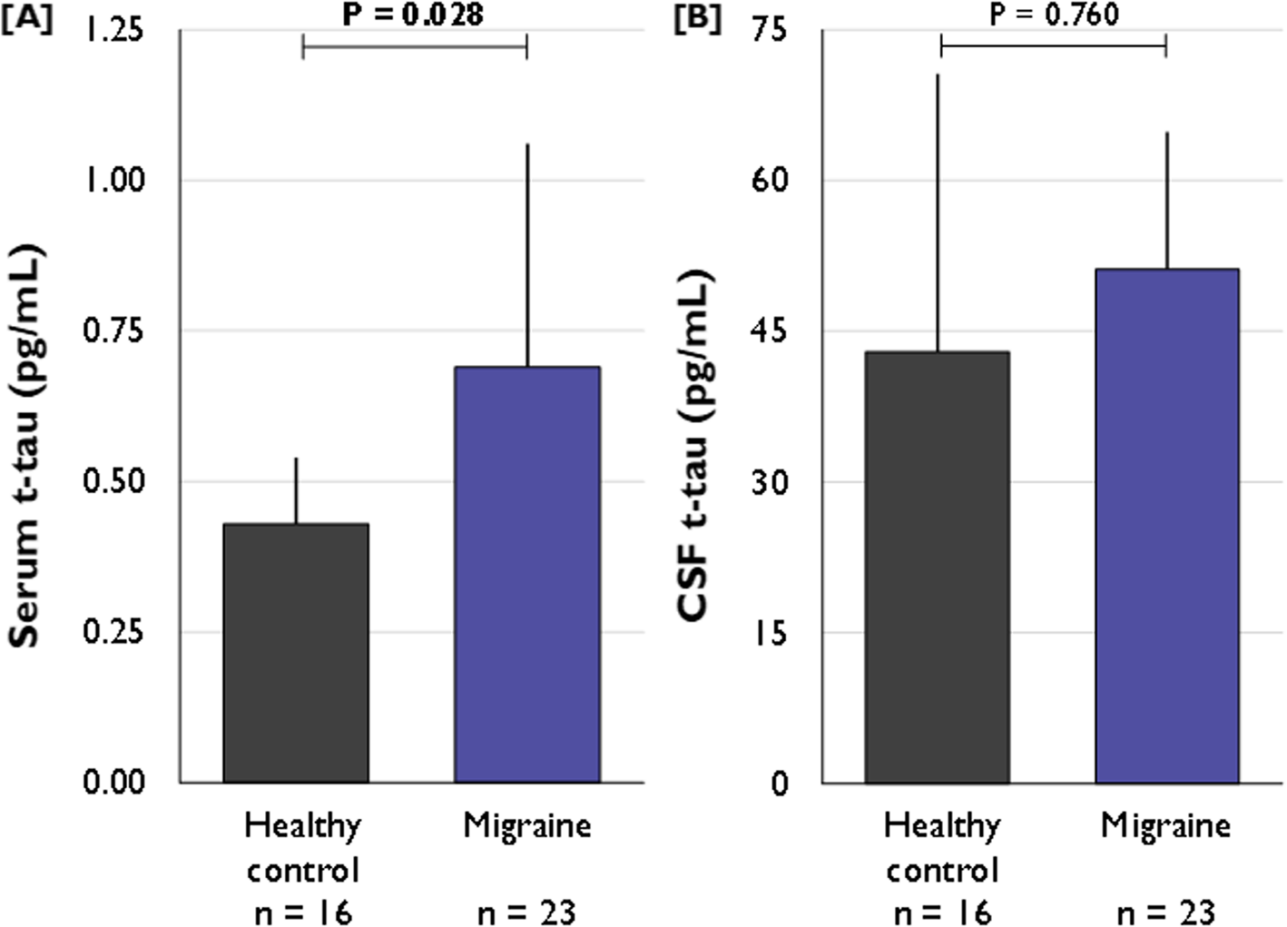
Serum (**A**) and CSF (**B**) t-tau concentration in migraine patients and controls. Legend *p*-values for between-group difference as estimated with the Independent-Samples Mann–Whitney U test for continuous variables. *p*-values < 0.05 are depicted in bold

In order to determine the source of t-tau changes, CSF and serum samples obtained during routine in-hospital diagnostic work-up of headache in patients eventually diagnosed with migraine (*n* = 23) and in patients presenting for exclusion of CNS pathology in psychosomatic disorders (controls; *n* = 16) were investigated using the same methodology. CSF samples were obtained by lumbar puncture and serum samples were drawn concomitantly. There was no difference in the albumin ratio (Qalb) between both groups (migraine 5.1 ± 2.0 × 10^–3^ vs. control 5.8 ± 2.7 × 10^–3^; *p* = 0.330). The mean age of the migraine group was 34.9 (22.4 to 42.7) years and included *n* = 20 (87%) females. For the control group the mean age was 27.0 (24.0 to 41.0) and included *n* = 12 (63%) females. Serum t-tau concentrations were elevated in migraine patients (*Md* = 0.69 pg/mL [0.39 to 1.06]) compared to samples taken in controls (*Md* = 0.43 pg/mL [0.33 to 0.54]); *p* = 0.028, *r* = 0.339), Fig. 3A. In contrast, CSF t-tau concentrations did not differ between migraine (*Md* = 51.17 [36.22 to 64.82]) and control samples (*Md* = 42.96 pg/ml [35.13 to 70.57], *p* = 0.760, *r* = 0.047), Fig. 3B.

## Discussion

This cross-sectional observational study assessed t-tau concentrations in serum samples of 227 participants with migraine and age- and sex-matched healthy controls. T-tau was elevated in patients with EM and CM compared to controls. Stratification for aura did not show any differences between patients with migraine with and without aura. CM patients without preventive medications had higher t-tau serum concentrations than HC, which could not be observed in CM patients with preventive medication. T-tau elevation was not observed in the CSF of migraine patients in comparison to controls. NfL, GFAP, and UCH-L1 were not different from controls and interictal migraine patients. These findings may point to a pathophysiological role of tau in migraine pathophysiology.

T-tau levels in EM patients were not different between migraine without and with aura. The latter is the clinical correlate of cortical neuronal depolarization [36]. The lack of a difference in both groups indicates that cortical activity is not relevant for tau release and implies that rather neuronal activity associated with pain, e.g. the trigeminal nerve or the ganglion is associated with tau release. However, our study is not suited to determine the origin of tau release in patients with migraine with certainty. Animal studies with a focus on the interplay of tau, PACAP and the PAC-1 receptor are needed to shed light on this question. Of note, no other markers associated with neuronal or glial damage were elevated in serum.

Tau is a microtubule-associated protein, which is involved in axonal transport as it stabilizes neuronal microtubules. Moreover, tau has a role in the formation, maintenance, and repetition of myelin by activating Fyn-kinase rafts. Only a few studies link tau protein, neuropeptides, and inflammation. Most recently, the small molecule CGRP receptor antagonist BIBN-4096, a drug that successfully aborts migraine attacks, reduced neuroinflammation in an experimental model of Alzheimer´s disease [23]. Tau pathology is a key component of this disease [37]. Tau was also elevated in inflammatory peripheral neuronal diseases such as Guillain-Barré syndrome as recently described [23, 38]. Interestingly, tau concentrations were significantly higher in inflammatory neuropathies than in non-inflammatory neuropathies, which indicates a pathophysiological link between tau protein and inflammation. In inflammatory neuropathies, tau elevation was seen along with a rise of neurofilament light chain (NfL) levels, which confirms structural damage. Inflammation in migraine is thought to be independent of structural damage. As inflammation and neuropeptide release has been described in migraine in the meninges and trigeminal ganglion (for review see Edvinsson, 2019), these structures could also be relevant for t-tau elevation in this disorder [36]. Markers associated with cell damage such as NfL, GFAP or UCHL-1 were normal in serum samples in this study. This observation is in line with our aforementioned hypothesis of neuro-inflammation and t-tau release and a rather functional role of tau in migraine. In line, in tau knock-out mice the neuronal response to noxious stimuli was reduced while the response to tonic painful stimuli was increase along enhanced evoked c-fos expression in the dorsal horn [39]. The latter observation supports a functional role of tau in pain conditions.

The estimated area under the curve (AUC) indicates that serum t-tau concentration could distinguish between patients with migraine and healthy controls in two-thirds of cases (*p* < 0.001). However, the low AUC (0.662) suggests an inadequate ability to discriminate between individuals with and without migraine. Therefore, the utility of serum t-tau levels as a diagnostic biomarker needs to be explored in future studies. More specifically, it remains to be determined whether t-tau levels can differentiate between different primary headaches.

At this stage, we cannot determine the clinical significance of tau in migraine. It is also worth noting that t-tau levels in migraine are significantly lower than in e.g. inflammatory neuropathies, brain trauma or neurodegenerative disorders. However, CGRP and PACAP levels in peripheral blood in migraine patients are also in the picogram range and nevertheless play a crucial role in migraine pathophysiology [40, 41]. A possible role of tau in migraine pathophysiology and the relationship between CGRP, PACAP, or the PAC-1 receptor with tau needs to be determined in future studies.

Tau is not different between EM and CM in this study indicating that migraine frequency is not a determining factor for tau levels. We show a moderate negative correlation of tau levels over time as determined from the last migraine day before blood withdrawal. The lack of a difference might be explained by relatively low tau levels in this study and the lack of accumulation of tau with increasing number of attacks. Whether pain intensity or the duration of migraine episodes play a role for elevated tau levels in blood remains to be determined.

The different t-tau concentrations observed in CM patients with prophylaxis compared to CM patients without prophylactic medication use is unlikely to be caused by lower number of MHD (mean 12.8 vs 17.0, respectively) since the number of MHD of EM patients is much lower (mean 7.2). It is possible that prophylactic medication reduces tau levels by other mechanisms than reducing headache frequency. A longitudinal study is necessary to provide evidence for any causality.

Alcohol consumption differed between migraine patients and controls in this study. Lower alcohol consumption in migraine patients has been observed before [42]. Accumulating evidence indicates that alcohol consumption leads to elevated t-tau concentrations [43]. In our cohort, no effect of alcohol consumption on serum t-tau concentrations was observed, nor did the correction for alcohol use affect the results (data not shown). This might be explained by the fact that none of our participants are considered heavy drinkers.

A strength of our study is the large sample size, which provides accurate and robust findings. Our strict in- and exclusion criteria helped to select patients and controls in a consistent, reliable, uniform, and objective way to avoid confounding or biasing factors. Another strength is the method of sample analyses. The single-molecule array (Simoa®) technology is an ultrasensitive method using antibodies, which is superior to the enzyme-linked immunosorbent assay (ELISA) technology. Therefore, our measurements are more accurate and precise than those from ELISA.

This study also has limitations. First, tau elevation in serum is not specific for any disease. The different types of headache diaries (paper/electronic) led to missing information on headache intensity and the precise duration of migraine attacks in hours. We did not check if patients had an attack immediately after blood collection and therefore, we cannot exclude the possibility that the patient was in the prodromal migraine phase. Based on the fact that premonitory symptoms are typical central CNS phenomena and that we found a significant tau increase in the periphery during attacks, we believe that an influence of a premonitory migraine stage on these findings is improbable.

In summary, this is the first report of t-tau elevation in a primary headache disorder. Patients with migraine have elevated t-tau levels in blood serum in the interictal state, but not in CSF compared to age- and sex-matched controls. Our findings indicate that tau is derived from a peripheral source in migraine. Future studies need to establish the precise role of tau in migraine.

### Supplementary Information

**Additional file 1: Supplementary Table 1.** Clinical and demographic characteristics of the subgroups of episodic and chronic migraine.

## Data Availability

We will make data available upon request to UR.
