# Supplementary material for: Serum tau protein elevation in migraine: a cross-sectional case–control study
Source: J Headache Pain. 2023 Sep 19;24(1):130. doi: 10.1186/s10194-023-01663-5 (PMC10507851; doi:10.1186/s10194-023-01663-5)
Supplement: Supplementary file 1 — Additional file 1: Supplementary Table 1. Clinical and demographic characteristics of the subgroups of episodic and chronic migraine. [file 10194_2023_1663_MOESM1_ESM.docx]

**Supplementary Table 1 Clinical and demographic characteristics of the subgroups of episodic and chronic migraine**

|  | **Episodic migraine** | | | **Chronic migraine** | | |
| --- | --- | --- | --- | --- | --- | --- |
|  | **EMO** | **EMA** | **P-value ^a^** | **CM-** | **CM+** | **P-value ^a^** |
|  | **n = 49** | **n = 43** |  | **n = 48** | **n = 45** |  |
| **General** |  |  |  |  |  |  |
| Age in years | 42.1 ± 10.3 | 41.3 ± 11.1 | 0.734 | 43.2 ± 12.0 | 47.2 ± 12.0 | 0.111 |
| Female, n (%) | 39 (79.6) | 34 (79.1) | 1.000 | 42 (87.5) | 38 (84.4) | 0.769 |
| BMI, mean ± SD | 24.2 ± 3.6 | 23.8 ± 4.0 | 0.644 | 23.5 ± 3.5 | 24.8 ± 4.5 | 0.125 |
| Ethnicity |  |  | 1.000 |  |  | 0.484 |
| Caucasian | 48 (98.0) | 43 (100) |  | 48 (100) | 44 (97.8) |  |
| Other | 1 (2.0) | 0 |  | 0 | 1 (2.2) |  |
| Alcohol consumption, n (%) |  |  | 0.804 |  |  | 0.958 |
| Never (rarely) | 19 (38.8) | 14 (32.6) |  | 26 (54.2) | 27 (60.0) |  |
| < Monthly | 8 (16.3) | 10 (23.3) |  | 3 (6.3) | 3 (6.7) |  |
| Monthly | 11 (22.4) | 11 (25.6) |  | 11 (22.9) | 8 (17.8) |  |
| Weekly | 11 (22.4) | 7 (16.3) |  | 8 (16.7) | 7 (15.6) |  |
| Daily | 0 | 1 (2.3) |  | 0 | 0 |  |
| Tobacco consumption, n (%) |  |  | 0.159 |  |  | 0.053 |
| Never | 39 (79.6) | 29 (67.4) |  | 37 (77.1) | 32 (71.1) |  |
| Past | 8 (16.3) | 7 (16.3) |  | 10 (20.8) | 6 (13.3) |  |
| Current | 2 (4.1) | 7 (16.3) |  | 1 (2.1) | 7 (15.6) |  |
|  |  |  |  |  |  |  |
| **Headache characteristics** |  |  |  |  |  |  |
| Total headache days | 7.4 ± 3.2 | 7.1 ± 3.4 | 0.695 | 17.0 ± 6.1 | 12.6 ± 7.9 | 0.005* |
| Total migraine days | 6.1 ± 2.8 | 6.2 ± 3.2 | 0.954 | 13.6 ± 6.4 | 10.8 ± 6.8 | 0.045* |
| Total acute medication days | 5.8 ± 2.7 | 4.9 ± 3.1 | 0.186 | 9.6 ± 6.5 | 7.5 ± 6.4 | 0.076 |
| Days since last headache attack | 3.5 ± 4.4 | 5.6 ± 6.5 | 0.237 | 1.3 ± 1.7 | 2.2 ± 3.7 | 0.714 |
| Days since last migraine attack | 4.6 ± 5.5 | 6.8 ± 7.3 | 0.270 | 1.9 ± 2.1 | 2.3 ± 4.0 | 0.354 |

BMI = body mass index, calculated as kg/m^2^ where kg is a person's weight in kilograms and m^2^ is their height in meters squared; SD = standard deviation; EMO = episodic migraine without aura; EMA = episodic migraine aura; CM- = chronic migraine without prophylactic treatment; CM+ = chronic migraine with prophylactic treatment.

Continuous values are expressed as mean ± SD.

^a^ p-value for between group difference, Chi^2^ test was used for categorical variables and Mann-Whitney Test for continuous variables.

* p-value < 0.05
